# The Role of Neurotropic B Vitamins in Nerve Regeneration

**DOI:** 10.1155/2021/9968228

**Published:** 2021-07-13

**Authors:** Simone Baltrusch

**Affiliations:** Institute of Medical Biochemistry and Molecular Biology, University Medicine Rostock, Rostock, Germany

## Abstract

Damage and regeneration naturally occur in the peripheral nervous system. The neurotropic B vitamins thiamine (B1), pyridoxine (B6), and cobalamin (B12) are key players, which maintain the neuronal viability in different ways. Firstly, they constantly protect nerves against damaging environmental influences. While vitamin B1 acts as a site-directed antioxidant, vitamin B6 balances nerve metabolism, and vitamin B12 maintains myelin sheaths. However, nerve injury occurs at times, because of an imbalance between protective factors and accumulating stress and noxae. This will result in the so-called Wallerian degeneration process. The presence of vitamins B1, B6, and B12 paves the way out to the following important regeneration by supporting the development of new cell structures. Furthermore, vitamin B1 facilitates the usage of carbohydrates for energy production, whereas vitamin B12 promotes nerve cell survival and remyelination. Absence of these vitamins will favor permanent nerve degeneration and pain, eventually leading to peripheral neuropathy.

## 1. Introduction

**Figure 1:**
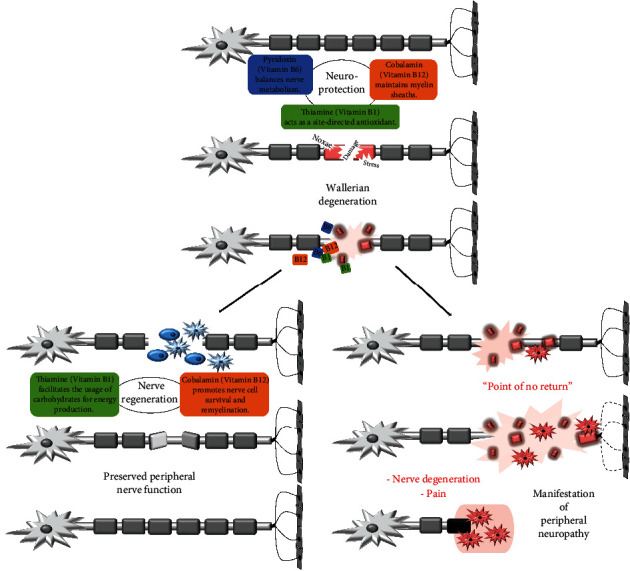
Neurotropic B vitamins can avoid manifestation of peripheral neuropathy (lower right) by directing the process of Wallerian degeneration to regeneration and remyelination (lower left).

Damage and regeneration naturally occur in the peripheral nervous system. If the balance of these two processes is disturbed, for example, by chronic diseases such as diabetes, and nerve regeneration is impaired, nerve damage can lead to peripheral neuropathy [1]. Nerve damage can have a single cause (e.g., traumatic compression as a result of acute crush injury or laceration as a result of transection injury), but in many cases (and almost always in the chronic course), it is a combination of reasons, such as laceration, traction, vibration, compression, ischemia, inflammation, alcoholism, metabolic (diabetes) or toxic noxae (chemotherapy), surgery, genetic causes, and deficiency in neurotropic B vitamins [1–6]. However, nerves are amazingly regenerative, and regeneration is even possible until approximately 50% of the fibers within a nerve (considered the “point of no return”) are damaged [1]. When a peripheral nerve is centrally injured, the surrounding nonnerve cells initiate the so-called Wallerian degeneration (Figure 1). In this process, the degradation of axon parts (fragmentation) is regarded as a key event that already occurs within one to a few days after the injury. Subsequently, at the site of injury, macrophages are recruited to carry away myelin and dead cells, and the distal stump degenerates. Furthermore, the nonnerve cell responses promote an environment that supports the regeneration of axons over the following months [7, 8]. It has been suggested that certain B vitamins support this nerve regeneration process (Figure 1). In particular, the vitamins B1 (thiamine), B6 (pyridoxine), and B12 (cobalamin) are mentioned in this context. Those are also called “neurotropic” vitamins because of their important functions in the nervous system [9]. Although it is not fully understood how they support the process, numerous animal studies provided evidence for the effect at the histological or molecular level in recent decades. Due to demographic trends, neurodegenerative diseases such as peripheral neuropathy are becoming increasingly important [10] and so will possible treatments.

This is clearly illustrated by diabetes mellitus, which according to the International Diabetes Federation currently affects 463 million people worldwide. Neuropathy is one of the most frequent late complications of this disease [11, 12]. The degeneration process of the nerves does not start with manifestation of diabetes mellitus, but already with impaired glucose tolerance [13]. By the time clinical symptoms of diabetic neuropathy appear, significant and irreversible nerve loss has often already occurred (Figure 1, “point of no return”) [11–13]. It can be speculated that by the time a reduction in nerve conduction velocity of myelinated A*δ* nerve fibers is diagnosed, neuropathy has already existed progressively for years, and treatment comes too late. In contrast, the diagnostic method of confocal microscopy of the eye's cornea, which has been increasingly used in recent years, allows noninvasive detection of changes in the thin C nerve fibers [14–18]. Until now, peripheral nerve fiber density could only be detected invasively by skin punch and subsequent immunofluorescence staining [19]. Independent studies have shown that the loss of skin innervation in diabetic polyneuropathy is preceded by a significant reduction in the nerves of the subepithelial plexus of the cornea [20, 21]. Thus, therapeutic concepts must also be readdressed from the perspective of improved diagnostics. The authors from a double-blind, randomized, controlled study concluded that neurotropic B vitamins represent a starting point in the treatment of diabetic polyneuropathy [22]. In addition to a benefit in the patients' subjective paresthesias, an improvement was also observed in objectively assessable parameters, such as a decrease in the vibration perception threshold at the second metacarpal and metatarsal bones and an increase in conduction velocity in the peroneal nerve [22]. Other human studies [23–27] and animal experiments [28–30] confirmed the benefit of continuous administration of vitamins B1, B6, and B12 with respect to diabetic neuropathy.

However, to date, there is no recent review article available focusing explicitly on the nerve-regenerating function of B vitamins. Therefore, systematic literature searches were performed to compile the actual evidence from animal studies, provide a current update on this topic for the individual vitamins B1, B6 ,and B12 as well as their combination, and try to derive biochemical explanations.

## 2. Strategy of Literature Search

First, systematic literature searches in PubMed (https://www.ncbi.nlm.nih.gov/pubmed) for articles that had been published through June 2020 using search term combinations of “vitamin B,” “thiamine,” “pyridoxine,” or “cobalamin” with “nerve regeneration” were conducted. Due to the low number of search results and because it was also aimed to include foreign-language articles, no filters for language, periods, etc. were used. These searches added up to only 101 results of which the majority dealt with vitamin B12. To receive additional evidence on vitamins B1 and B6 by further PubMed searches, search term combinations of “thiamine OR vitamin B1[Title/Abstract]” or “pyridoxine OR vitamin B6[Title/Abstract]” with “nerve role OR nerve function” (filter: “other animals”) were applied. With the 318 additional results, a total of 419 results (including duplicates) from PubMed were observed, of which 383 results remained after removing duplicates. Six additional relevant articles from manual searches in Google Scholar and Google were added. Thereafter, abstracts or, in uncertain cases, full texts were screened. Finally, a total of 26 relevant original articles in the field of experimental animal research were included (Table 1). The exclusion criteria included lack of relevance, redundancy (e.g., secondary publication), human studies, and reviews. However, human studies and reviews (very few relevant articles) in certain cases were used for a more comprehensive overview. Two of the listed full text articles also had to be translated into English because the original articles were published in Russian and Japanese.

## 3. Results and Discussion

As shown in Table 1, several animal studies provide evidence for a role of neurotropic B vitamins (B1, B6, and B12) in the process of nerve regeneration. Despite extended searches for vitamins B1 and B6, most evidence is still related to vitamin B12. Even for the combination of vitamins B1, B6, and B12, comparatively fewer studies were available.

### 3.1. Vitamin B1

Vitamin B1 (thiamine) plays a key role as a coenzyme in the carbohydrate metabolism, which is the main energy supply for nerve fibers. Thiamine pyrophosphate is essential for feeding pyruvate to the oxidative energy metabolism, eventually resulting in adenosine triphosphate (ATP) production [29, 31]. In addition, several studies suggest that it acts as a site-directed antioxidant, thereby protecting nerves from oxidative damage [32].

Two animal studies on vitamin B1 about nerve regeneration were identified. One of them showed a regeneration-related effect but both showed neuroprotection (Table 1). Stracke et al. were able to show that vitamin B1 protected peripheral nerves against damage induced by hyperglycemia in living rats, particularly when given early in the course of diabetes. Precisely, they revealed that nerve conduction velocity increased substantially in vitamin B1-treated diabetic rats after three months administration compared with untreated diabetic controls, which was attributed to reduced formation of advanced glycation end-products (AGEs) [29]. Moreover, Song et al. found that vitamin B1 positively influenced nerve excitability of rat neurons after chronic compression of dorsal roots, i.e., it led to improved signal transmission and at the same time reduced hyperexcitability [33], indicating a regenerative effect. These results are supported by an in vitro study from Geng et al., demonstrating that vitamin B1 promoted survival of cultured rat brain neurons in high-density cell culture [34].

Furthermore, a small study from 1976 on patients suffering from the thiamine deficiency disease beriberi provided histological evidence for a regenerative effect of thiamine. Because of Schwann cell clusters with regenerated axons (some even remyelinated), the authors concluded that vitamin B1 stimulated the regeneration of axonal or Wallerian degeneration that takes place in beriberi [35].

Altogether, vitamin B1 (thiamine) plays a pivotal role in the process of nerve regeneration: in nerve cells, it facilitates the usage of carbohydrates for energy production and protects them against oxidative stress, resulting in normalized pain sensation and reduced hyperexcitability.

### 3.2. Vitamin B6

Vitamin B6 (pyridoxine) is essential for the amino acid metabolism. Hence, vitamin B6 assures the metabolism of neurotransmitters (e.g., GABA and serotonin), which are indispensable for signal transmission in the nervous system, and is associated with the glutamate release. Vitamin B6 may inhibit glutamate release by suppressing presynaptic voltage-dependent Ca2+ entry and protein kinase C activity [36]. In addition, vitamin B6 can—by increasing GABA synthesis—balance the activity of excitatory glutamatergic neurons [37]. It is also needed for the synthesis of sphingolipids—essential constituents of the nerve-surrounding myelin sheath [38].

The one relevant animal study for vitamin B6 rather demonstrates neuroprotection than nerve regeneration (Table 1). In a rat model, vitamin B6 counteracted the nerve-damaging effect of excessive glutamate release, which is an important mechanism of neuronal damage in certain neurological diseases [36]. Clinical evidence on a regenerative function, however, can be derived from a human study in patients with carpal tunnel syndrome (compression injury), in which vitamin B6 treatment increased the conduction velocity of sensory nerves and thus reduced clinical symptoms [39]. Experiments in monkeys showed that vitamin B6 prevented neuronal death in the retina after whole brain ischemic damage from death [40]. However, because of the general function of vitamin B6 in metabolism, highlighting a specific role in nerve regeneration remains difficult.

In conclusion, vitamin B6 (pyridoxine) plays a key role in neurotransmitter synthesis, inhibits the release of neurotoxic glutamate, and restores of sensory nerve function.

### 3.3. Vitamin B12

As mentioned previously, the evidence for a nerve-regenerating function of vitamin B12 (cobalamin) is strong, and 15 experimental animal studies are available (Table 1). For instance, experiments in rats revealed that it promoted myelin formation and reduced Wallerian degeneration responses [41, 42] and were, similar to vitamin B1 and B6, also believed to have a neuroprotective effect [42] (Figure 1). Cobalamin is essential for the folate dependent methionine cycle. If the amount of B12 does not cover the demand (which is significantly increased during nerve regeneration and myelin formation), not only can important proteins (e.g. the myelin basic protein) not be generated but also homocysteine accumulates and promotes oxidative stress and damage [41–45]. This may aggravate Wallerian degeneration and delay or even prevent the progress of regeneration. While the important role of vitamin B12 in axon regeneration because of microtubule stabilization has been only shown in several diseases of the central nervous system (CNS), another effect, namely, the endoplasmic reticulum (ER) stress level is also probably decisive for the peripheral nervous system (PNS) [43–45]. Especially in the case of a nerve injury concomitant with inflammation, ER stress increases and exceeds self-protective levels, resulting in neuronal death [43–45].

Using vitamin B12 for nerve regeneration in mice after sciatic nerve injury, Yuan et al. demonstrated that it did not only significantly promote functional recovery but also increased the number of myelinated fibers, the diameters of myelinated fibers and axons, and the lamellae number [46]. Similar results have been obtained by Gan et al. who proved a dose-dependent increase of myelin thickness in mice. After the left sciatic nerve of the mice was surgically cut, the animals were subcutaneously treated with vitamin B12 in phosphate-buffered saline (PBS) or only PBS for 12 weeks [47]. In this study, the myelin sheath in regenerated myelinated nerve fibers was significantly thicker in high-dose vitamin B12-treated animals than in the placebo/saline group [47].

Besides effects on myelination, there is cumulating evidence for nerve recovery. Improved nerve terminal and axonal regeneration [48, 49] and increased nerve fiber density [50, 51] were reported. The therapeutic effect of vitamin B12 involves the upregulation of multiple neurotropic factors [47, 52], namely, nerve growth factor (NGF) and brain-derived neurotrophic factor, or possibly enhanced protein metabolism [53] which are thought to promote the nerve survival and regeneration. However, little is known about how acute or chronic vitamin B12 deficiency, on the one hand, and long-term supplementation, on the other, affects the formation of neurotrophic factors in the PNS. In the adult PNS, the availability of limited quantities of neurotrophic factors in a target-derived manner is essential for neuronal health. Studies suggest that vitamin B12 induces a positive balance between neurotrophic factors rather than just their expression [54]. As shown for the CNS, but most likely also true for the PNS, vitamin B12 may keep tumor necrosis factor *α* down and interleukin 6 and NGF upregulated, thereby promoting nerve regeneration after injury [54, 55]. Another study showed that vitamin B12 treatment delayed the onset of diabetic peripheral neuropathy via upregulation of the neural insulin growth factor-1 gene expression [29].

Two studies with rats strengthen the proposal that vitamin B12 fulfils its nerve-regenerating function by inhibiting apoptosis of damaged neurons, by affecting the methylation cycle and thereby kinases promoting neurite outgrowth and neuronal survival and by creating an environment that supports recovery [56, 57]. Dexamethasone [52] as well as cholecalciferol [58], synergistically with vitamin B12, showed significantly greater improvement of the nerve function than vitamin B12 alone. However, both effects were only demonstrated in a small cohort of animals and hitherto not proven elsewhere.

It should be also noted that positive effects of vitamin B12 on the recovery of the motor and sensory function and nerve regeneration, especially myelination, could also be shown for a new local application, namely, an electrospun nanofiber sheet incorporating methylcobalamin with local delivery [59]. While the majority of these studies examined the nerve-regenerating effect in the PNS, the results may also be transferable to the CNS [43, 60]. Wu et al. recently showed that vitamin B12 treatment in mice after traumatic brain injury rescued neurological function, stabilized microtubules, and promoted remyelination and myelin repair [43].

In summary, there is convincing evidence that vitamin B12 (cobalamin) particularly holds a nerve-regenerating role and promotes nerve cell survival, remyelination, and the maintenance of myelin sheaths, whereby improvement or even a complete cure of nerve function with physiological sensory nerve conduction velocity is achieved.

### 3.4. Combination of Vitamins B1, B6, and B12

Although vitamins B1, B6, and particularly B12 already show nerve-regenerating effects individually, one may assume that combining them enables synergies [61] and thereby supports nerve regeneration even more effectively. Indeed, this assumption was confirmed by Jolivalt et al. who demonstrated that the combination of vitamins B1, B6, and B12 restored sensory nerve function in rats with experimental diabetes more effectively than the individual B vitamins and did so in a dose-dependent manner [28]. Beyond that, in vitro nerve outgrowth of murine dorsal root ganglia was stronger with a combination of high-dose vitamins B1, B6, and B12 compared to combinations in which only one of the three vitamins was high dose [62]. In addition, accelerated nerve regeneration by the combination of vitamins B1, B6, and B12 was suggested from other studies [63, 64] which, however, were too small to show significant results. A recently published study investigated the effect of vitamin B therapy in the regeneration of experimental crush peripheral nerve injury to rats and appeared to contradict this finding and attributed vitamin B12 alone or in combination with B1 and B6 a stronger nerve-regenerating effect than vitamin B1 or B6 alone [65]. However, while the authors specified the doses of the individually administered vitamins (B1/B6/B12 180/180/1 mg/kg/day), they did not break it down for the combination (described as a total dose of 20 mg/kg/day). It must therefore be assumed that the combination contained less vitamins B1 and B6 than the single vitamin injections. In addition, it must be taken into consideration that the basic vitamin supply differed between the studies due to varied animal feed, making a comparison more difficult. Also, it is worth mentioning that vitamin B12 can be stored to some extent in the liver; although, it is a water-soluble vitamin. Thus, the study confirms that vitamin B12 is more important for nerve regeneration than vitamins B1 and B6 alone, but it is not sufficient to demonstrate a superiority of vitamin B12 over the combination.

Further histological evidence for the nerve-regenerating effect of the combination was provided by Becker et al. who applied cold damage to the saphenous nerve of 50 rabbits. They subsequently treated 25 rabbits with high-dose vitamins B1, B6, and B12 for 21 days while the remaining rabbits received placebo. In fact, the B vitamin combination significantly increased the number of regenerated axons, especially of myelinated fibers [66]. Moreover, a vitamin B complex containing vitamins B1, B2, B3, B5, B6, and B12 attenuated the increase in nerve and muscle nuclear density observed after injury to the femoral nerve and its target muscle in rats [67]. Another study using the same vitamin B complex demonstrated reduced local inflammation after peripheral nerve injury [68].

## 4. Summary and Conclusion

All of the three highlighted B vitamins may create the necessary environmental conditions for successful nerve regeneration, each of which via individual modes of action (concluded in Figure 1). Vitamin B1 essentially facilitates the energy production needed for the process and acts as a site-directed antioxidant, while vitamin B6 is vital for neurotransmitter synthesis and for inhibiting the release of neurotoxic glutamate [29, 32, 40]. Vitamin B12, on the other hand, largely promotes nerve cell survival and is strongly and directly involved in remyelination and the maintenance of myelin sheaths [41, 42, 46, 47, 56]. However, to elucidate molecular mechanisms, prove nerve-regenerating functions, and investigate neuroprotection, further experimental in vitro and in vivo studies with the individual B vitamins and the combination are needed.

## Figures and Tables

**Table 1:** Included experimental animal studies on the nerve-regenerating effect of neurotropic B vitamins.

Reference	Studied B vitamin(s)	Species	Main findings/conclusion	Nerve-regenerating effect shown?
Studies on vitamin B1 (thiamine) only				
Song 2009 [33]	B1	Rat	Thiamine suppressed thermal hyperalgesia, reduced hyperexcitability, and lessened alterations of sodium currents in injured dorsal root ganglion neurons.	Yes
Stracke 2001 [29]	B1	Rat	In comparison to water-soluble thiamine nitrate, early administration of liposoluble vitamin B1 (benfotiamine) prevented formation of advanced glycation end-products and functional nerve damage to a much higher degree than when given later during experimental diabetes.	n/a, neuroprotection

Studies on vitamin B6 (pyridoxine) only				
Yang 2009 [36]	B6	Rat	Vitamin B6 inhibited glutamate release from rat cortical synaptosomes through the suppression of presynaptic voltage-dependent Ca^2+^ entry and protein kinase C activity.	n/a, neuroprotection

Studies on vitamin B12 (cobalamin) only				
Albay 2020 [58]	B12	Rat	B12 attenuated sciatic nerve injury. B12 and cholecalciferol synergistically improved functional and histopathological nerve healing.	Yes
Horasanli 2017 [48]	B12	Rat	Vitamin B12 promoted functional recovery, improved axonal regeneration, and attenuated edema and myelin sheath degeneration after sciatic nerve injury.	Yes
Wang 2015 [56]	B12	Rat	Vitamin B12 had an antiapoptotic effect and possibly promoted nerve regeneration by inhibiting the apoptosis of damaged neurons and creating conditions for the recovery of nerve function.	n/a
Gan 2014 [47]	B12	Mouse	High-dose vitamin B12 promoted functional recovery of nerves (sciatic nerve) after peripheral nerve injury. It also promoted morphological recovery, possibly by upregulation of neurotrophic factors.	Yes
Romano 2014 [69]	B12	Rat	Vitamin B12 treatment accelerated reepithelization and corneal reinnervation after mechanical injury (removal of corneal epithelium).	Yes
Tamaddonfard 2014 [42]	B12	Rat	High-dose vitamin B12 increased functional recovery and promoted peripheral nerve regeneration by reducing Wallerian degeneration responses after tibial nerve crush injury.	Yes
Sun 2012 [52]	B12	Rat	Dexamethasone and vitamin B12 synergistically promoted peripheral nerve repair after sciatic nerve injury through the upregulation of brain-derived neurotrophic factor expression and were more effective in combination than both of the treatments alone.	Yes
Jian-bo 2010 [30]	B12	Rat	Vitamin B12 delayed the onset of diabetic peripheral neuropathy via upregulation of neural insulin–like growth factor 1 gene expression, particularly together with good glycemic control.	Yes
Liao 2010 [41]	B12	Rat	Vitamin B12 facilitated functional recovery, enhanced motor end plate innervation, and augmented the diameters and myelin thickness of regenerated axons following end-to-end neurorrhaphy.	Yes
Okada 2010 [57]	B12	Rat	High-dose vitamin B12 promoted neurite outgrowth and neuronal survival and increased extracellular signal-regulated kinases and Akt signaling after sciatic nerve injury. It also promoted functional recovery of nerves.	Yes
Yuan 2010 [46]	B12	Mouse	Vitamin B12 promoted functional recovery and histological regeneration of the injured sciatic nerve and its target muscle after sciatic nerve injury.	Yes
Watanabe 1994 [50]	B12	Rat	High-dose vitamin B12 promoted functional recovery and increased nerve fiber density, numbers of small- and medium-sized myelinated fibers, and fiber diameters in acrylamide-induced peripheral neuropathy.	Yes
Yamazaki 1994 [49]	B12	Mouse	Vitamin B12 promoted regeneration of degenerating nerve terminals in gracile axonal dystrophy, possibly by acting both on motorneurons and Schwann cells.	Yes
Mikhaĭlov 1987 [51] (translated from Russian into English)	B12	Rat	Vitamin B12 accelerated the reinnervation of skeletal muscles after experimental mechanical damage.	Yes
Yamatsu 1976 [53] (translated from Japanese into English)	B12	Rat	Vitamin B12 enhanced protein metabolism in Schwann cells at the initial stages of axonal regeneration after sciatic nerve injury and thereby possibly facilitated axonal regeneration.	n/a

Studies on vitamin B complex (including at least B1, B6, B12) and possibly further B vitamins				
Al-Saaeed 2019 [65]	B1, B6, B12	Rat	Results confirmed that vitamin B12 played an essential role in neuronal regeneration through myelination of the injured nerve. It promoted nerve regeneration better than the other B vitamins and better than the combination.	Yes
Ehmedah 2019 [68]	B1, B2, B3, B5, B6, B12	Rat	Vitamin B complex treatment attenuated local inflammation after peripheral nerve injury.	Yes
Nedeljković 2017 [67]	B1, B2, B3, B5, B6, B12	Rat	Treatment with vitamin B complex (B1, B2, B3, B6, B12) immediately after injury and reconstruction of the peripheral motor nerve improved recovery of the injured nerve. Vitamin B complex attenuated muscle atrophy and the increase in nerve and muscle nuclear density, which was observed after injury of the femoral nerve and its target muscle.	Yes
Altun 2016 [63]	B1, B6, B12	Rat	Tissue levels of vitamin B complex and vitamin B12 varied with progression of crush-induced sciatic nerve injury. Supplementation of these vitamins in the acute period may help accelerate nerve regeneration.	n/a
Jolivalt 2009 [28]	B1, B6, B12	Rat	In experimental diabetes, repeated daily treatment with vitamin B complex (B1, B6, B12) ameliorated tactile allodynia and formalin-evoked hyperalgesia in a dose-dependent manner and improved sensory nerve conduction velocity. None of the individual B vitamins was as effective as the combination in restoring nerve function (nerve conduction velocity).	Yes
Fujii 1996 [62]	B1, B6, B12	Rat, mouse	Vitamin B complex (B1, B6, B12) promoted neurite outgrowth and effectively treated acrylamide-induced neuropathy. It played an important role in growth and repair of nerve fibers.	Yes
Becker 1990 [66]	B1, B6, B12	Rabbit	Vitamin B complex (B1, B6, B12) increased the number of regenerated axons, especially of myelinated fibers, and decreased the occurrence of degradation products in the degenerated nerve section after cold lesion of the *N. saphenous* compared with placebo.	Yes
Stotzem 1988 [64]	B1, B6, B12	Rat	Vitamin B complex slightly increased nerve regeneration after sciatic nerve injury but the difference vs. control was not statistically significant.	Small, statistically nonsignificant difference

n/a: not applicable.

## Data Availability

The literature that support the conclusions of this review are within Table 1.
